# In-depth comparison of somatic point mutation callers based on different tumor next-generation sequencing depth data

**DOI:** 10.1038/srep36540

**Published:** 2016-11-22

**Authors:** Lei Cai, Wei Yuan, Zhou Zhang, Lin He, Kuo-Chen Chou

**Affiliations:** 1 Bio-X Institutes, Key Laboratory for the Genetics of Developmental and Neuropsychiatric Disorders (Ministry of Education), Shanghai Key Laboratory of Psychotic Disorders (No.13dz2260500), Shanghai Jiao Tong University, Shanghai, 200030, China; 2 Gordon Life Science Institute, Boston, Massachusetts, 02478, USA; 3 Institute of Biliary Tract Disease, Xinhua Hospital, Affiliated to Shanghai Jiao Tong University School of Medicine, Shanghai, 200092, China; 4 Women’s Hospital School Of Medicine Zhejiang University, Hangzhou, 310006, China; 5 Center of Excellence in Genomic Medicine Research (CEGMR), King Abdulaziz University, Jeddah, 21589, Saudi Arabia

## Abstract

Four popular somatic single nucleotide variant (SNV) calling methods (Varscan, SomaticSniper, Strelka and MuTect2) were carefully evaluated on the real whole exome sequencing (WES, depth of ~50X) and ultra-deep targeted sequencing (UDT-Seq, depth of ~370X) data. The four tools returned poor consensus on candidates (only 20% of calls were with multiple hits by the callers). For both WES and UDT-Seq, MuTect2 and Strelka obtained the largest proportion of COSMIC entries as well as the lowest rate of dbSNP presence and high-alternative-alleles-in-control calls, demonstrating their superior sensitivity and accuracy. Combining different callers does increase reliability of candidates, but narrows the list down to very limited range of tumor read depth and variant allele frequency. Calling SNV on UDT-Seq data, which were of much higher read-depth, discovered additional true-positive variations, despite an even more tremendous growth in false positive predictions. Our findings not only provide valuable benchmark for state-of-the-art SNV calling methods, but also shed light on the access to more accurate SNV identification in the future.

Rapid advancement in next-generation sequencing (NGS) technologies has brought about a more comprehensive dissection of genomic variations at a more affordable expense than ever before. NGS has recently become a routine to reveal subtle genetic variations in cancer genomes^1^,^2^,^3^. By applying NGS on tumor-normal tissue pairs, multiple research projects, including the Cancer Genome Atlas (TCGA), the International Cancer Genome Consortium (ICGC), and the Cancer Genome Project (CGP), have characterized critical genomic variations involved in the occurrence and development of cancer. It is widely considered that certain ancestral cells, which gain enough somatic mutations to cause dysregulations in genome stability/repair and cell proliferation, acquire selective advantages, reproduce abnormally into cell populations with a catalog of mutations at diverse frequencies, and initiate and develop tumor^4^. Among the different types of genetic variations, somatic single nucleotide variants (SNVs), the most frequent type in cancer genomes, are single nucleotide mutations only occurring in tumor samples not in normal samples of a patient^5^. Due to their extensive existence and remarkable biological functions, somatic SNVs have attracted much attention in cancer research, which are supposed to contain crucial “driver” mutations in oncogenesis and tumor progression^6^,^7^.

Since different algorithms using different information have their own strengths and weaknesses, it has been suggested to use the consensus outputs from multiple algorithms to obtain a more reliable prediction for a missense variant^8^,^9^,^10^. Accordingly, although identifying somatic SNVs from NGS data is quite straightforward and simple theoretically, it still encounters considerable challenges, such as: sample degradation, polymerase chain reaction (PCR) artifacts, inadequate coverage, position-specific error rate and read alignment errors. Moreover, additional issues must be addressed for somatic SNV calling in sample pairs, such as distinguishing somatic mutations from germline variations, and handling genetic heterogeneity and tissue impurity. To meet these challenges and reduce false predictions, scientists have adopted distinct strategies and statistical models and developed a considerable number of software tools or algorithms for detection of somatic mutations, such as the currently most popular somatic SNV callers: Varscan, SomaticSniper, Strelka, and MuTect2^11^,^12^,^13^,^14^,^15^,^16^.

Most of these algorithms directly compare tumor-normal pairs at every locus^17^, label sites with minor allele frequencies significantly higher in tumor samples compared to those in normal samples as somatic variants, and meanwhile exclude putative germline variants and loss of heterozygosity (LOH) events from the final candidate sets. Although each of the new tools has different updated versions after years of improvement, they still have poor consensus in realistic scenarios^18^,^19^. Moreover, due to incomplete experimental evaluation and lack of an established gold-standard method for massive somatic SNV calling featuring with highest sensitivity and specificity, their relative merits in real applications are largely unknown.

Although a few efforts have been made using real or simulated sequencing data to compare and/or benchmark different callers^20^,^21^, the drawbacks of these comparisons or benchmarks may seriously hinder the thorough assessment of SNV calling methods. First, although Sanger sequencing is widely viewed as a reliable experimental verifying method, it is not only expensive and laborious but also inappropriate for alleles of low fraction (e.g., <20%)^22^,^23^. It may also restrict the scale of validation to only 100~300 sites and brings forth potential bias in assessment, since NGS may generate hundreds to thousands of candidates for a single tumor-normal tissue pair. Moreover, the false negatives are difficult to be validated by this method. Second, although simulated data are widely used for their easy access, low cost, and clear constitution of positives and negatives, several common artifacts are beyond current simulation yet, such as: the non-random distribution of variants, incomplete reference genome, and copy number variations (CNVs)^24^. Since simulation datasets are collections of synthetic reads based on simple generative models while real datasets are much more complex and harder to call variation on, they may not truly tell the same story as real sequencing data^25^. Third, as for the use of indirect properties of mutation calls instead of direct validation in these studies, multiple metrics of the prediction sets must be weighed to estimate the performance of each program rather than simply counting overlaps between different methods or calculating the average read depths. Furthermore, most previous studies are undertaken on fairly limited quantity of data, which may further impair the trustworthiness of these studies. Meanwhile, targeted sequencing with ultra-deep read depth has been applied rapidly during the past few years. Typical whole exome sequencing (WES) provides only up to ~60X read depth and works unsatisfactorily in calling low fraction alleles. Using targeted gene panel sequencing that allows read depth of more than 500X, researchers are able to unveil mutations with rare allelic frequency, which is of great significance to solve tumors substantial heterogeneity. However, till now, few studies have been conducted to compare the performance of these somatic SNV detection tools based on different tumor NGS depth data, especially the ultra-deep targeted sequencing (UDT-Seq) data.

Considering these constraints, we set out to evaluate the performance of most popular somatic SNV callers (Varscan^12^, SomaticSniper^14^, Strelka^13^, and MuTect2^15^) on real NGS data^26^. The WES data and UDT-Seq data were used as input to each caller separately, and outputs were carefully gaged by multiple metrics. Then, we analyzed the merits and shortcomings of UDT-Seq compared to WES in view of somatic SNV detection. Furthermore, based on the current in-depth analysis, Cake^27^, a pipeline for the integrated analysis of somatic SNVs in cancer genomes, was utilized to evaluate the combination of diverse methodologies.

## Results

### Calling of four somatic SNV callers

We applied the four somatic SNV callers (Varscan, SomaticSniper, Strelka and MuTect2) on the WES (32 samples) and target sequencing data (54 samples) from totally 57 matched tumor-normal tissue pairs. Sequencing summary is available in Supplementary Table S1. In order to follow the original intention of developers, all tools were executed with default parameters and filters recommended by the caller manuals. High-confidence calls produced by each caller then underwent further analysis.

**Figure 1:**
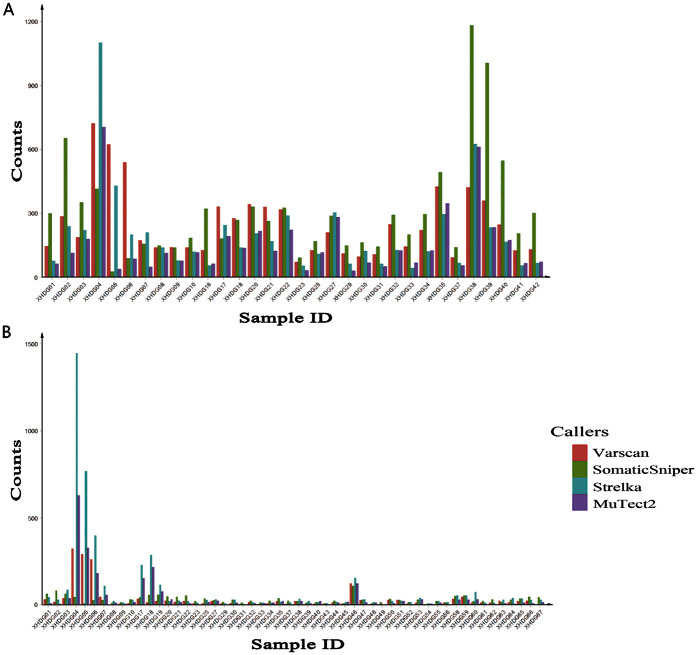
Counts of mutations detected by four sSNV callers in different patients based on WES (**A**) and UDT-Seq (**B**) data.

**Figure 2:**
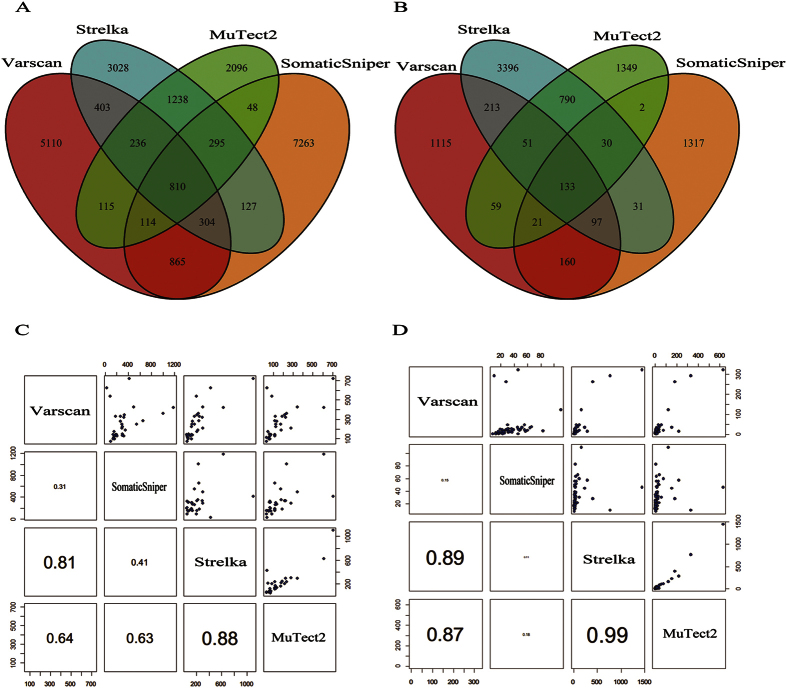
The relationship of mutations detected by four sSNV callers. Venn diagrams of mutations based on WES (**A**) and UDT-Seq (**B**) data illustrate the overlaps between mutation candidate sets. Scatter-plot of mutation candidate sets from each sample based on WES (**C**) and UDT-Seq (**D**) data demonstrate the correlation between size of mutations detected by different callers: each dot in the upper panel represents the number of mutations detected from each tumor-normal tissue pair, and the lower panels display the Pearson correlation coefficients between the two callers with font size proportional to the correlations.

On the same sample, callers varied dissimilarly in the amount of SNV candidates, and on the same sample and caller, different depth caused different amount of candidates, as shown in Fig. 1. For example, among four callers Strelka obtained the largest candidate set in samples XHDG04, XHDG05, XHDG07 and XHDG27 under exome sequencing, while in a number of extra samples besides XHDG04, XHDG05, XHDG07 and XHDG27 under UDT-Seq. In total, Varscan and SomaticSniper detected more SNV candidates (7957 and 9826) based on WES data, whereas Strelka and MuTect2 acquired more candidates (4741 and 2435) based on UDT-Seq data (Supplementary Fig. S1). The overlaps between the four sets of SNV candidates are illustrated in Fig. 2. Although four different callers showed quite low consistency (only 20.7% of all mutations were detected by two or more callers within WES data and 22.4% within UDT-Seq data), Strelka and MuTect2 shared the largest fraction of common candidates (2579 of 8814 within WES data and 1004 of 6172 within UDT-Seq data) and had the highest correlation in the number of candidates obtained from different samples (0.88/0.99 for WES/UDT-Seq, respectively).

### Evaluation of somatic SNV callers by multiple metrics of candidate sets

**Figure 3:**
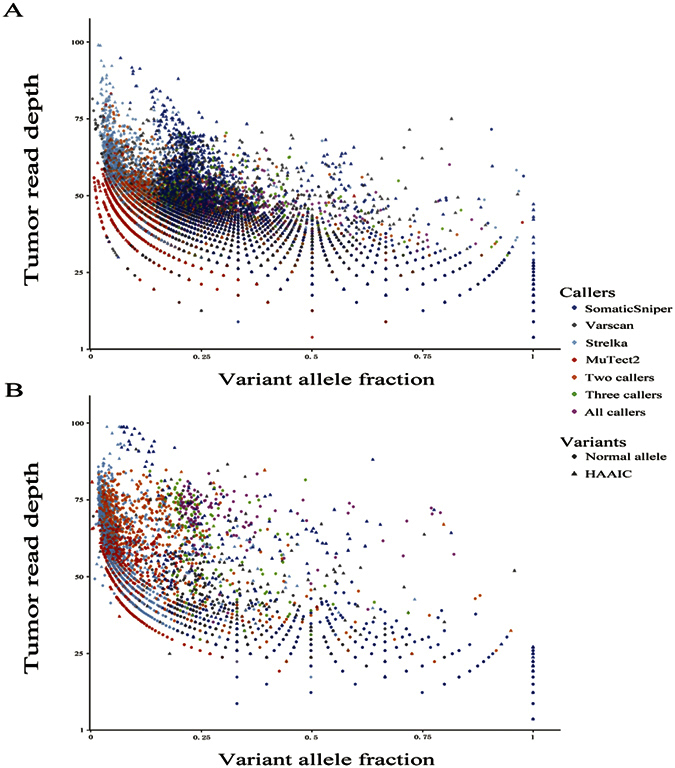
The depth-VAF scatter plot of SNV candidates in WES (**A**) and UDT-Seq (**B**).The Y-axis of each graph means the read depth of a variant in the tumor tissue, while the X-axis means the VAF of each SNF candidate. Triangles means SNV candidates have HAAIC. The color means SNV candidates detection methods.

In current study, three internal features of candidate sets were utilized in the identification of false predictions. One is the high-alternate-alleles-in-control (HAAIC) variants, which are excessively unreliable and are considered as likely false-positives. Strelka and MuTect2 revealed advantage in eliminating these counterfeits, particularly in WES (5.9% and 4.6%, respectively, Table 1). Interestingly, variants with high read depths found in tumor samples are a large fraction of HAAIC variants (Fig. 3). The other is the strand bias, which provides a very probable source of false-positives during calling somatic SNVs. Except for a small fraction of sites with obvious extreme strand bias in Varscan’s call list (3.6% and 4.4%), other call sets exhibited satisfyingly low percentage of calls with strand bias (Table 1). The last is the somatic SNVs occurring in normal samples of other patients more than twice observations. Since somatic SNV calls with more than two hits in normal tissues had a higher share of dbSNP entries in both WES and UDT-Seq (p < 0.001, Supplementary Table S2), somatic SNV calls with more than two observations in normal controls tended to be germline polymorphisms, and thus are unreliable. Strelka and MuTect2 showed low percentages (~10% in WES and ~40% in UDT-Seq) of mutations with more than two observations in controls, while the Varscan and SomaticSniper callers obtained a larger rate of questionable SNVs (50.8% and 71.3% in WES and 42.7% and 67.0% in UDT-Seq, respectively, Table 1).

We also took public databases (dbSNP Human Build 138^28^ and COSMIC v70^29^) as references for evaluating caller performance. Candidates present in dbSNP are regarded as putative false positives caused by germline polymorphisms or sequencing errors^19^,^30^. Although it is not adequate to assign a single site to putative false positives solely by its appearance in dbSNP, the proportion of dbSNP entries in a candidate set implies its unreliability. Similarly, sites present in COSMIC provide a reference to estimate the ratio of true-positive SNVs^31^,^32^. Somatic SNVs generated by each caller were annotated if they exist in COSMIC or dbSNP database. A counting of annotated sites is listed in Table 1. SomaticSniper returned much higher dbSNP rate (~70%) than any other tools in both groups, suggesting its inferior ability in false-positive control. For WES and UDT-Seq, MuTect2 demonstrated an excellent performance with the lowest dbSNP rate (14.9% and 38.4%, respectively) and the highest COSMIC rate (7.1% and 13.2%, respectively) among four callers. Strelka had relatively higher dbSNP rate and lower COSMIC rate compared with MuTect2 (15.9% dbSNP presence and 4.8% COSMIC presence), even in UDT-Seq (40.6% dbSNP presence and 8.2% COSMIC presence), where it gained much more calls than any other tools. The dbSNP and COSMIC annotation information was mapped to the depth-VAF scatter plot (Supplementary Fig. S2).

A Sanger sequencing validation by the previous study^24^ was also employed in our benchmark. Albeit not eligible to serve as a criterion for comparison of callers due to its biased choice of candidates, the Sanger sequencing data make a valuable reference for estimating the sensitivity of callers. The results indicated that Strelka gained a very close number of validated calls (47/58 validated candidates detected in WES and 60/64 in UDT-Seq) to that of MuTect2, while a much lower level of validation was made by Varscan and SomaticSniper (~20 candidates in WES and ~30 in UDT-Seq, Table 2).

### Cake analysis

As different callers adopt distinct algorithms and strategies in somatic SNV calling, an intuitive strategy to improve the methodology in somatic mutation calling would be utilizing different callers’ candidate sets and generating high-confidence collections of somatic variants^32^,^33^. Here, Cake^27^ was used for combination and filtering of SNV candidate sets separately generated by Varscan, SomaticSniper, Strelka, and MuTect2. Finally, 2691 variants from WES data and 227 ones from UDT-Seq data were retained after Cake analysis, respectively. Compared to those given by individual callers, Cake calls had a higher rate of COSMIC entries (6.7% and 22.0% within WES and UDT-Seq data, respectively) and lower rates of dbSNP entries (10.4% and 19.4%, respectively) and HAAIC calls (~ 4%), albeit at the cost of sensitivity and size of candidate sets (Table 3).

To visualize the SNV produced by Cake, we marked out those variants in the depth-VAF scatter plot (Supplementary Fig. S3). Cake managed to discover SNVs at widespread depths and VAF, except for those with very low depth of reads in tumor samples or low variant allele proportions. Although of limited absolute amount, the SNV candidates produced by Cake proved to be with higher likely true-positives and lower likely false-positives.

### Comparison between WES and UDT-Seq

**Figure 4:**
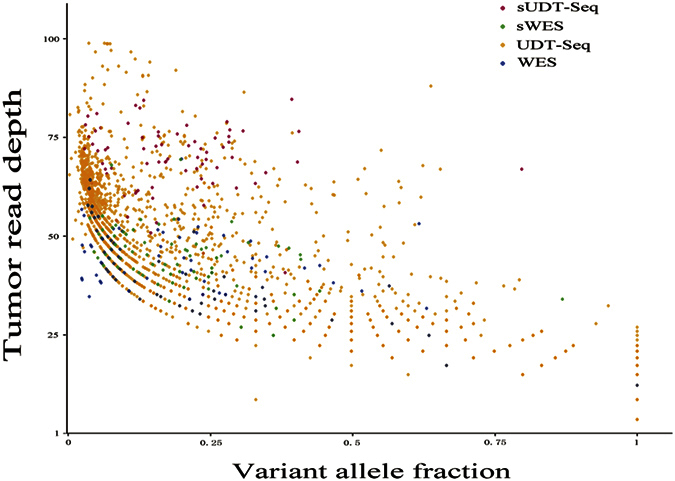
The intersection of SNVs obtained from WES and UDT-Seq data. The dots demonstrate SNV candidates from 23 tumor-normal tissue pairs undergoing both WES and UDT-Seq. sUDT-Seq: SNVs detected within UDT-Seq are shared with those within WES; sWES: SNVs detected within WES are shared with those within UDT-Seq; UDT-Seq: SNVs uniquely detected within UDT-Seq data; WES: SNVs uniquely detected within WES data.

To assess the effect of read depth on somatic SNV discovery, we compared SNV candidates located in gene regions covered by UDT-Seq from WES data to those from UDT-Seq data in the 23 samples that were sequenced by both methods. Totally, 2175 UDT-Seq and 182 WES SNV candidates were taken into comparison. UDT-Seq provided candidates in much larger amount and much wider spread of distribution on depth-VAF scatter plot than WES, and the sites agreed by both UDT-Seq and WES had relatively high read depths on each sequencing method (Fig. 4). UDT-Seq had SNV candidates with a higher rate of COSMIC and dbSNP entries (5.6% and 49.1%, respectively) than WES (4.9% and 23.6%, respectively), and produced more treacherous SNV candidates (49.7% somatic SNVs with more than two observations in control and 20.3% HAAIC calls; Table 4).

## Discussions

The current bottleneck of NGS lies in data management and analysis due to the complex variants detection process from huge sequencing data^34^. Till now, there are many mutation callers, such as: Varscan, SomaticSniper, Strelka and MuTect2, etc. The complexities within somatic SNV calling are caused by both dissimilar mathematical models adopted by different callers and the biological divergence underlying each sample. Here, we found that the same analysis tool might exhibit sharp fluctuations among samples besides different callers might vary differently on the same sample. For instance, somatic SNV candidate sets share impressive overlap for some samples (XHDG04, XHDG22, XHDG27, XHDG32, XHDG35, etc.), which is in agreement with some reports^21^,^35^, but not for other samples, which is also consistent with some studies^19^. Therefore, any attempts for a persuasive somatic SNV caller benchmark should be implemented on enough quantities of samples. Moreover, new versions of somatic SNV callers have appeared very soon. Different settings of parameters in variation detection and filtration of different tool version could make even larger divergences than the internal diversities of algorithms do. Thus, only comparing the number of somatic SNVs provided by each tool is not quite meaningful. In this study, we focused on the intersection and unique parts of multiple candidate sets acquired from various samples, tools and sequencing depths, and compared multiple properties of somatic SNV candidate sets acquired from the last versions of these four popular somatic SNV callers, i.e., Varscan, SomaticSniper, Strelka and MuTect2. Furthermore, tumors have significant heterogeneity and tumors from different tissues may have different gene alterations. Thus, the default settings suggested by each caller developers were used in the current study to obtain ideal results for individual caller.

Usually, the presence of somatic SNV in COSMIC provides a measure for the true-positive, while dbSNP entries, strand-biased sites, observation in normal controls, and HAAIC calls provides a measure for the false-positive. Multiple callers employ widely different strategies to handle these false-positive characters. For example, Strelka discards strand-biased somatic SNV, whereas MuTect2 and Varscan leave them to users’ disposal^17^. However, since Ti/Tv ratio is variable and dependent on the cancer types^20^, it is not analyzed, in our comparison. Although MuTect2 returned fewest candidates, it had excellent capability in both control of false calls and discovery of potential true positives. MuTect2 refers to dbSNP database to aid its classifier in discrimination between germline and somatic variants, and turns out to be efficacious. Strelka had the second-best performance close to that of MuTect2. Interestingly, Strelka predicted second least somatic SNVs in WES data, much less than SomaticSniper and Varscan did, whereas it gave most predictions in UDT-seq. Among the four callers, MuTect2 and Strelka have fairly similar performance: they have the largest consensus in somatic SNV candidate sets, similarly high sensitivity in detecting low-allelic-fraction somatic SNVs, and integrated effective post-calling filters, which were also verified by other studies^22^{Roberts, 2013 #37}. Varscan yielded medium specificity and sensitivity. Although Varscan was not efficient in low-frequency mutations detection, it exhibited advantage in discovering somatic SNVs with relatively high frequencies, which makes it as a beneficial supplement of MuTect2 and Strelka. SomaticSniper returned the largest quantity of mutations in WES data, a majority of which were questionable predictions with a plethora of strand-biases, somatic SNVs detected in normal samples and dbSNP entries.

Furthermore, we also visualized the distribution of somatic SNV candidates on depth-VAF scatter plot. It was observed that the methods showed different preference for somatic SNV discovery and common calls detected by multiple methods are mainly located central of the scatter plot. In detail, MuTect2 is able to discover unique somatic SNVs at low depth and low VAF; Strelka favored calling somatic SNVs with higher read depth and lower VAF than other three callers; somatic SNVs obtained from Varscan preferred moderate tumor read depth and VAF; and SomaticSniper had a wide spread of somatic SNVs except those with both low depth and low VAF.

Compared to other two callers, both Strelka and MuTect2 preferred variants with low VAF, but they varied in choice of tumor depths. These divergences root in the underlying mechanisms of algorithms and the enormous heterogeneity of candidate sites. Accordingly, when benchmarking different callers, they are not exactly homogeneous and comparable. Some of callers may outperform the others, but this would not guarantee that they have a better performance in all regions of the depth-VAF scatter plot. The fact that the best-performing callers may return considerable fractions of suspicious calls in their high-confidence outputs was found in both our study and previous studies^19^. Thus, more sophisticated mutation-calling tools are expected in the future to be equipped with multiple differentiated models and algorithms, intelligently adopting different strategies in consideration of the variant sites’ sequencing properties (read depths, read quality, strand bias, variant allele fraction, etc.).

Owing to a lack of a priori knowledge on samples and function of callers, we accepted nearly all default parameters and filters of every caller, which are considered best fitting for most of the situations and reflecting the developers’ original intention. However, it should never be ignored that the tuning of somatic SNV detection parameters and the customizing filtration tremendously affect the quality and reliability of outputs^19^,^22^. Since all callers tested by us presented quite numerous false-positives and false-negatives, appropriate caller tuning and application of additional filters might be necessary for practical cancer research. Moreover, additional filters beyond the default filtering process of callers, which concern about multiple metrics simultaneously in judgment, are vitally suggested, and much more comprehensive tests and in-depth understanding of callers are required for carefully benchmarked and optimized somatic point mutation discovery.

From the depth-VAF scatter plots, it could also be observed that somatic SNVs detected by more than two methods were located in a limited region, even when MuTect2 or Strelka, which could produce a large proportion of true-positive unique somatic SNV candidates, was included. This indicates that predictions generated by various methods disperse substantially, only considering consensus of them may be inadequate. Thus, Cake, a pipeline that automatically combines somatic SNV callers and conducts follow-up filtration, was used on both WES and UDT-Seq. somatic SNVs acquired by Cake showed larger fraction of COSMIC entries, much lower rates of dbSNP entries, and HAAIC calls, which indicated that Cake was able to improve the specificity and increase the reliability of somatic SNV calls obtained from different callers. Nevertheless, Cake may also lead to a sharp reduction in quantities of candidates, including those present in COSMIC database. Therefore, when applying this strategy, researchers should make it clear whether they are pursuing a handful of reliable mutations for further validation or a wide genomic landscape including mutations of moderate to low fraction.

Moreover, the current study also provides insights into the effect of read depth. Although disparate in read depths and region of coverage, WES and UDT-Seq have no essential difference in principle and technology. However, trade-offs between sensitivity and specificity deeply underlie the comparison between WES and UDT-Seq. Calling in UDT-Seq data yields somatic SNV candidates of much larger quantity and higher diversity in the depth-VAF scatter plot than that in WES data, whereas a similar percentage of true positive and higher percentage of false positive is also observed. Thus, adopting UDT-Seq and increasing the depth of sequencing may not always help somatic SNV calling: it provides researchers with much higher resolution at the price of shrunken sequencing regions, larger proportion of false-positives, and increasing difficulties in selection/ filtration of calls. Necessary optimization of the pipelines and parameters are required to control false-positive calls in UDT-Seq. Furthermore, some callers may vary their performance under different depths: MuTect2 and Strelka did more impressive jobs on UDT-Seq data than WES data, whereas Varscan had poorer function on UDT-Seq data than WES data. Consequently, a profound understanding and a clear goal in study design are crucial for the successful detection of the desired mutations. In cancer genome, there is a particular significance for high-quality detection of low frequency mutations, since researchers often desire to capture minor mutations out of the vast heterogeneity of cancer. Moreover, in cancer research practice, most tumor contents are of substantial impurity, inevitably diluting the mutations of interest. Thus, we would increase depths in tumor benefit discovery of low-fraction SNV while the total cost remains acceptable.

In conclusion, there is a substantial discordance among samples, methods, and depths of reads in somatic SNV calling. Through our analysis, multiple facts indicate that MuTect2 and Strelka are of superior performance, and combining the results of different callers helps increasing the reliability of candidates but sacrifices enormous amount of uniquely detected calls. Compared to WES, calling in UDT-Seq obtained remarkably more real variation for given regions, although false-positive predictions even outgrow real ones as read depths increase. Our analysis provides a detailed and visualized reference for somatic SNV identification in WES and UDT-Seq, as well as orientations for further technological progress.

## Methods

Sequencing reads of patients were obtained from our previous study^24^. A total of 57 Chinese patients with only gallbladder carcinoma were recruited for the NGS, 29 of which were for both WES and UDT-Seq. 3 patients were processed with only WES and 25 were with only UDT-Seq. Whole-exome enrichment was performed using the TruSeq Exome Enrichment kit (Illumina). Captured DNA libraries were sequenced with the Illumina HiSeq 2500 Genome Analyzer, yielding 200 (2 × 100) base pairs from the final library fragments. UDT-Seq included 283 genes in the target enrichment panel. Targeted gene enrichment was performed with the TruSeq Custom Enrichment kits (Illumina). The validation data using Sanger sequencing was also acquired from this study^24^.

All sequencing reads were trimmed and filtered using Trimmomatic^36^. Following is the alignment of resulting reads to hg19 reference genome with Burrows-Wheeler Aligner (BWA, http://bio-bwa.Sourceforge.net/). Then we used the Genome Analysis Toolkit (GATK)^37^ for base quality score recalibration, indel realignment, and duplicate removal, reads with quality below 20 were discarded. We did not remove duplicate reads during UDT-Seq data analysis since normal reads may be eliminated for the application under high read depths.

Four popular somatic SNV caller, i.e. Varscan, SomaticSniper, Strelka and MuTect2 (Supplementary Table S3) were run on above pre-processed sequencing data and with default parameters recommended by the developers. We set the somatic quality threshold of SomaticSniper to 30 (the author recommended 15–40). Raw call sets generated by Varscan and SomaticSniper were filtered by pipelines proposed by the developers, and those generated by Strelka and MuTect2 were processed with built-in post-calling filters for either tool. Calls from the sample XHDG04, XHDG05 and XHDG06 pairs were removed for the posterior analysis from the total UDT-Seq call sets since their outliers contain excessive calls than other samples (more than 10 fold) and may have undue weight effects.

To identify false SNV predictions, first, we marked out variants with alternate alleles called in their corresponding normal samples through following standards: having too many alternate allele reads (>5 for WES, and >15 for UDT-Seq) in normal tissue; alternate allele reads constituting more than 10% of the total reads in normal tissue; and alternate allele reads in normal tissue exceeding 20% of that in corresponding tumor tissue. These sites, which are referred to as “high-alternate-alleles-in-control (HAAIC) variants”, are excessively unreliable and are therefore considered as likely false-positives. Second, Strand bias, which was common in Illumina sequencing data^17^ and provides a very probable source of false-positives, was examined by Fisher’s exact test. Variants with Phred-scaled p-value above 30 were regarded as strand biased. Third, since a true somatic SNV of one patient could rarely be a germline polymorphism in other subjects, we used Unified Genotyper of GATK to genotype all somatic SNV candidate sites in normal samples and calculated the total count of variant alleles of each somatic SNV candidate site in all controls. Each somatic SNV candidate occurring in normal samples of other patients more than two observations was marked as false positives. To visualize the distribution of SNV candidates, depth-VAF scatter plot was drawn using R software version 3.1.2 (www.r-project.org/), in which the Y-axis means the read depth of a SNV candidate in the tumor tissue, while the X-axis means the fraction of reads carrying the variant alleles, i.e. variant allele fraction(VAF) of each SNF candidate. The read depth of a SNV candidate is calculated based on normalized depth according to the following equation:


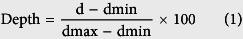


where d means the measured tumor read depth of a SNV, dmin means the minimum tumor read depth in a SNV candidate set, dmax means the maximum tumor read depth in a SNV candidate set.

Furthermore, SNV candidate sets were compared with dbSNP Human Build 138 and COSMIC v70, and calls were annotated for their appearance in these two databases. Since these two databases overlap which means that a small percentage of SNVs in dbSNP are somatic, candidates that are annotated by both COSMIC and dbSNP are only considered “COSMIC” but “non-dbSNP”. A total of 120 somatic SNVs by NGS were verified by Sanger sequencing. Excluding the loci that failed in PCR, 108 of 120 SNVs (90%) were successfully validated.

Cake was used for combining calls from multiple somatic mutation-callers. Four callers (SomaticSniper, Varscan, MuTect2 and Strelka)^38^ were applied by the program on pre-processed sequencing data(.bam files), and the post-processing module of Cake automatically sorted out somatic SNVs called by two or more methods, passing them to a further series of filtrations. Read depths and qualities were also extracted from the final outputs. We examined the outputs in dbSNP and COSMIC entries, strand bias and observation in controls.

To evaluate the effects of different sequencing depths on somatic SNV detection, we filtered candidate sets of WES data to keep only SNV candidates located within gene regions covered by UDT-Seq. Then, these subsets of SNV candidates underwent a comparison to those obtained from UDT-Seq.

Significant progresses have been achieved recently in developing various genome analysis tools^39^,^40^,^41^,^42^,^43^, which have been successfully used to analyze various important problems in genome analysis. It has not escaped our notice that these powerful new computational tools are very useful for in-depth studies in this area and our efforts are being underway.

## Additional Information

**How to cite this article**: Cai, L. *et al*. In-depth comparison of somatic point mutation callers based on different tumor next-generation sequencing depth data. *Sci. Rep.* **6**, 36540; doi: 10.1038/srep36540 (2016).

## Supplementary Material

Supplementary Information

## Figures and Tables

**Table 1:** Multiple features used for gauging call sets produced by four sSNV callers.

Whole Exome Sequencing or WES (32 samples)				
Method	Varscan	SomaticSniper	Strelka	MuTect2
Total variants	7957	9826	6441	4952
HAAIC variants	2156 (27.1%)	2319 (23.6%)	380 (5.9%)	228 (4.6%)
Variants with strand bias^a^	286 (3.6%)	20 (0.2%)	39 (0.6%)	10 (0.2%)
Variants with >2 observations in controls	4042 (50.8%)	7006 (71.3%)	715 (11.1%)	560(11.3%)
Variants present in dbSNP	3724 (46.8%)	6976 (71.0%)	1024 (15.9%)	738 (14.9%)
Variants present in COSMIC^b^	374 (4.7%)	452 (4.6%)	309 (4.8%)	352 (7.1%)
Targeted sequencing (51 samples)				
Total variants	971	1699	2128	1295
HAAIC variants	179 (18.4%)	227 (13.4%)	298 (14.0%)	191 (14.7%)
Variants with strand bias^a^	43 (4.4%)	12 (0.7%)	62 (2.9%)	13 (1%)
Variants with >2 observations in controls	415 (42.7%)	1139 (67.0%)	811 (38.1%)	480 (37.1%)
Variants present in dbSNP	396 (40.8%)	1151 (67.7)	864 (40.6%)	497 (38.4%)
Variants present in COSMIC^b^	58 (6.0%)	91 (5.4%)	174 (8.2%)	171 (13.2%)

^a^Phred-scaled p-value > 30.

^b^Variants present in both COSMIC and dbSNP are considered only with COSMIC entries since dbSNP holds a small fraction of somatic mutation.

**Table 2:** Sanger sequencing validation for SNV candidates.

Item	Varscan	SomaticSniper	Strelka	MuTect2	SNVs for Sanger resequencing
WES	23 (35.4%)	21 (32.3%)	47 (72.3%)	58 (89.2%)	65
Targeted sequencing	34 (50.0%)	31 (45.6%)	60 (88.2%)	64 (94.1%)	68

**Table 3:** Comparison between candidates identified by the four callers and those by Cake.

Method	Total variants	COSMIC*	dbSNP	HAAIC	Mean depth	Mean VAF
WES	22052	1125 (5.1%)	11004 (49.9%)	4896 (22.2%)	53.21	0.33
WES after Cake	2691	180 (6.7%)	280 (10.4%)	105 (3.9%)	35.16	0.28
Targeted sequencing	4642	281 (6.1%)	2505 (54.0%)	842 (18.14%)	192.77	0.29
Targeted sequencing after Cake	227	50 (22.0%)	44 (19.4%)	9 (4.0%)	367.06	0.28

**Table 4:** SNV Candidates detected by four callers based on WES and UDT-Seq data in 23 samples.

Methods	Total sites	COSMIC^b^	dbSNP	SNVs with >2 observations in controls	HAAIC^c^
WES^a^	182	10 (5.6%)	43 (23.6%)	60 (33.0%)	27 (15.1%)
Targeted sequencing	2175	680 (31.3%)	1067 (49.1%)	1081 (49.7%)	441 (20.3%)

^a^WES produces SNV candidates located in gene regions covered by UDT-Seq.

^b^Variants present in both COSMIC and dbSNP are considered only with COSMIC entries.

^c^HAAIC is abbreviation for High-Alternate-Alllele-In-Control.
