# Supplementary material for: In-depth comparison of somatic point mutation callers based on different tumor next-generation sequencing depth data
Source: Sci Rep. 2016 Nov 22;6:36540. doi: 10.1038/srep36540 (PMC5118795; doi:10.1038/srep36540)

In-depth comparison of somatic point mutation callers based on different tumor next-generation sequencing depth data

Lei Cai[1,4*], Wei Yuan[1], Zhou Zhang[1,2], Lin He[1,3], Kuo-Chen Chou[4,5]

1. Bio-X Institutes, Key Laboratory for the Genetics of Developmental and Neuropsychiatric Disorders (Ministry of Education), Shanghai Key Laboratory of Psychotic Disorders (No.13dz2260500), Shanghai Jiao Tong University, Shanghai 200030, China.
2. Institute of Biliary Tract Disease, Xinhua Hospital, Affiliated to Shanghai Jiao Tong University School of Medicine, Shanghai 200092, China.
3. Women's Hospital School of Medicine Zhejiang University, Hangzhou 310006, China.
4. Gordon Life Science Institute, Boston, Massachusetts 02478, United States of America.
5. Center of Excellence in Genomic Medicine Research (CEGMR), King Abdulaziz University, Jeddah 21589, Saudi Arabia.

Supplementary Information

Table S1. Sequencing summary of WES and targeted sequencing

Items	WES	Targeted sequencing
Sample number	32*2	54*2
Reads passed filtration	71064825±14074367	12972646±2980762
Reads mapped (%)*	98.5 (97.4-99.6)	99.7 (99.6-99.7)
Capture efficiency (%)*	59.6 (57.9-61.3)	62.2 (59.2-65.4)
Mean depth	50.4±10.2	366.2±79.2
≥ 1 coverage (%)*	95.9 (95.4-96.2)	98.7 (98.6-98.8)
≥ 4 coverage (%)*	93.4 (92.1-94.0)	N/A
≥ 8 coverage (%)*	91.0 (89.2-91.7)	N/A
≥10 coverage (%)*	N/A	96.4 (95.5-96.9)
≥20 coverage (%)*	80.7 (77.9-83.0)	N/A
≥50 coverage (%)*	N/A	67.8 (64.9-72.3)
≥200 coverage (%)*	N/A	89.5 (85.8-91.2)

*Data are presented as mean±sd or median(0.25-0.75quartile)

Table S2. Enrichment of SNVs calls with more than twice observations in controls with dbSNP database

Item	WES		Target sequencing	
Method of calls	Calls with ≤ 2 observation in controls	Calls with > 2 observation in controls	Calls with ≤2 observation in controls	Calls with > 2 observations in controls
dbSNP	7940	3069	1948	557
non-dbSNP	9213	1830	1852	285

Table S3. Algorithm tools for sSNVs detection within NGS data

Tools	Version	URL	Remark	Release date
MuTect2	2.0	https://software.broadinstitute.org/gatk/guide/tooldocs/org_broadinstitute_gatk_tools_walkers_cancer_m2_MuTect2.php	Sensitive detection of low allelic-fraction	Nov. 2015
SomaticSniper	1.0.5.0	http://gmt.genome.wustl.edu/2015/07/16/somatic-sniper_v1.0.5.0_released.html	High computational efficiency	Jul. 2015
Strelka	1.0.14	ftp://ftp.illumina.com/v1-branch/v1.0.14/	Clean outputs through stringent filtering	Jul. 2014
VarScan	2.4.0	http://dkoboldt.github.io/varscan/	Sensitive detection of high-quality sSNVs	Aug. 2015
MuTect2	2.0	https://software.broadinstitute.org/gatk/guide/tooldocs/org_broadinstitute_gatk_tools_walkers_cancer_m2_MuTect2.php	Sensitive detection of low allelic-fraction	Nov. 2015

Figure S1. Mutations detected by each caller based on WES (A) and UDT-Seq (B) data. Mutations are classified by the number of callers detection.

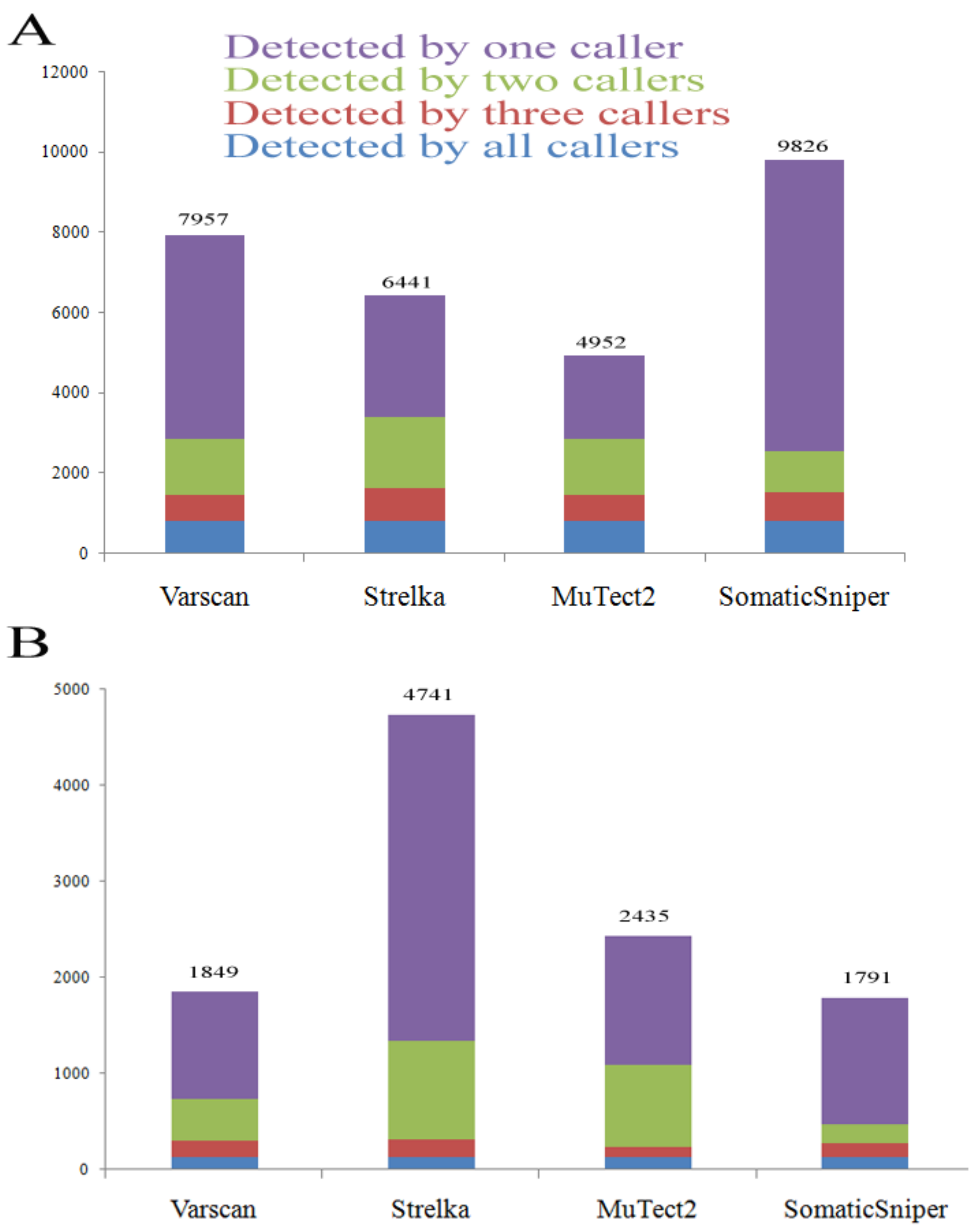

Figure S2. Somatic mutation candidates found in COSMIC and dbSNP database. A: WES, B: UDT-Seq. Variants present in both databases are classified into COSMIC ones.

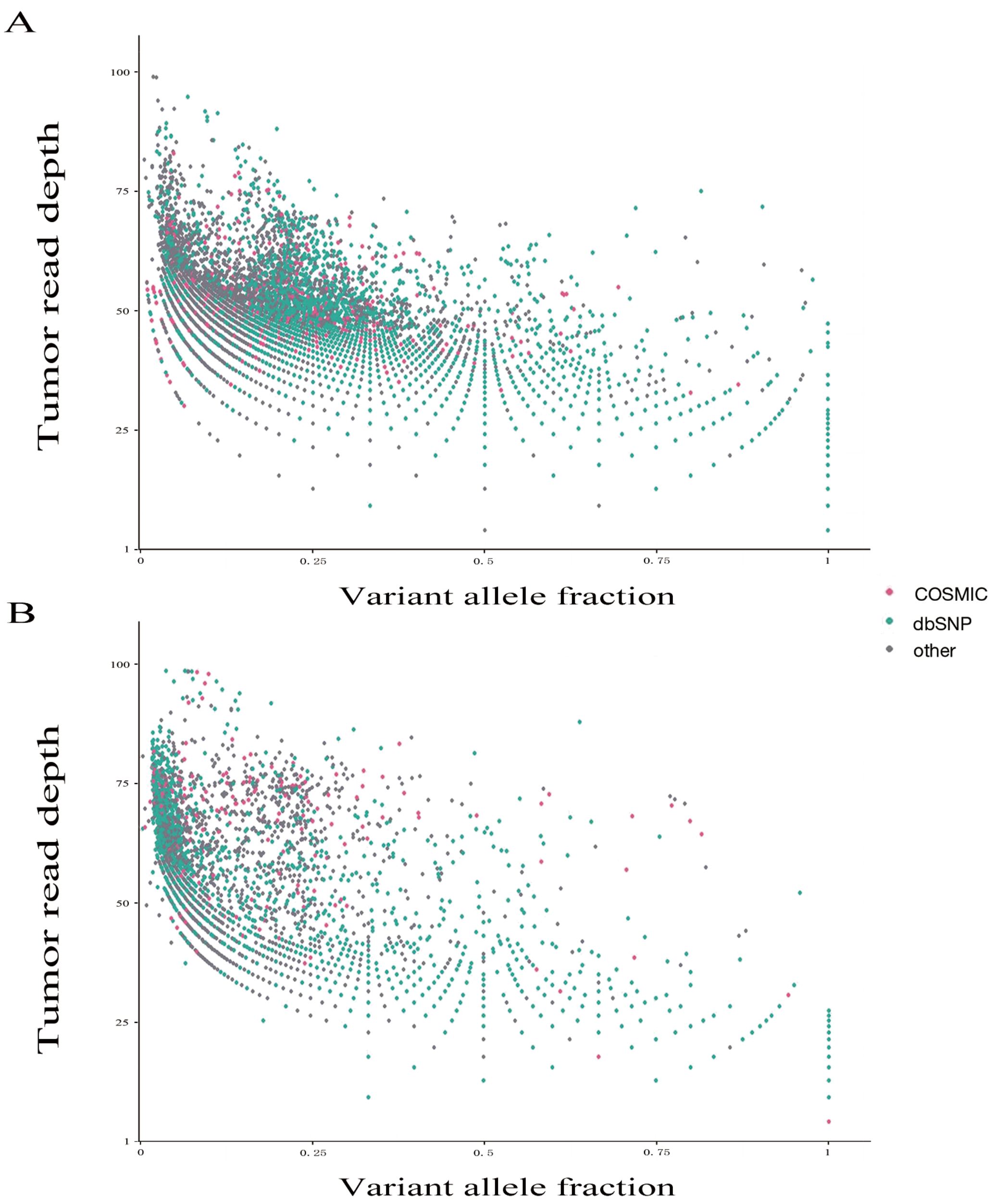

Figure S3. Candidates detected by Cake. A: WES; B: UDT-Seq. Only Cake calls that overlap with the collection of candidates by the four callers were shown in the figure.

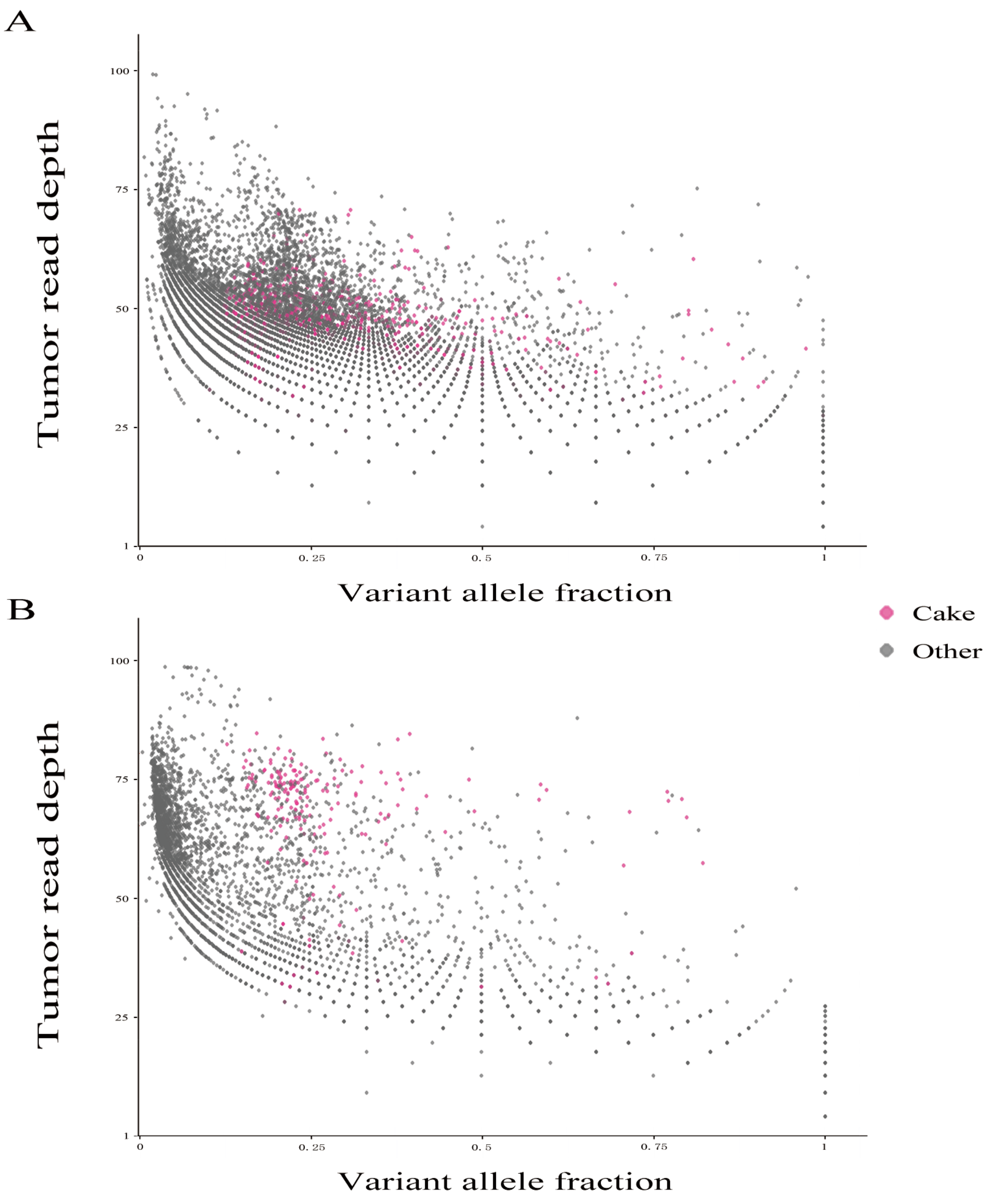

Supplement: Supplementary Information [file srep36540-s1.pdf]
